# Beyond relapses: How BTK inhibitors are shaping the future of progressive MS treatment

**DOI:** 10.1016/j.neurot.2025.e00602

**Published:** 2025-05-08

**Authors:** Laura R. Naydovich, Jennifer L. Orthmann-Murphy, Clyde E. Markowitz

**Affiliations:** Department of Neurology, Perelman School of Medicine, University of Pennsylvania, Philadelphia, USA

**Keywords:** Bruton’s tyrosine kinase (BTK) inhibitors, Progressive multiple sclerosis (PMS), Neuroinflammation, Compartmentalized inflammation, Clinical trials

## Abstract

Multiple sclerosis is a biologically and clinically heterogenous inflammatory demyelinating disease, driven by relapsing and progressive mechanisms, all individuals experiencing varying degrees of both. Existing highly effective therapies target peripheral inflammation and reduce relapse rates but have limited efficacy in progressive MS due to poor blood-brain barrier penetration and inability to address neurodegeneration. Bruton's tyrosine kinase (BTK) inhibitors represent an emerging therapeutic class offering a novel mechanism targeting BTK, which is expressed by both B cells and myeloid cells, including microglia within the CNS. Pre-clinical, Phase II, and Phase III clinical trials have demonstrated promising results in modulating progressive disease in both relapsing and non-relapsing MS patients. In contrast, the evidence regarding impact on relapse biology remains mixed and somewhat inconclusive. This review highlights gaps in current therapeutic strategies, examines the latest evidence for the efficacy and safety of BTK inhibitors in MS, and explores the future landscape of MS treatment.

## Introduction

Multiple sclerosis (MS) is a chronic, immune-mediated, biologically and clinically heterogenous central nervous system (CNS) disease. Differing theories exist as to how and where MS “begins,” but evidence suggests that various environmental factors, combined with genetic susceptibility, disrupt the normal checks and balances of the immune system, leading to injury targeting myelin in the brain and spinal cord [1].

Two key drivers of MS-related damage are relapsing disease and non-relapsing progressive disease, though most to all people with MS (pwMS) have elements of both and exhibit varying manifestations along the clinical spectrum. The reasons behind biological and clinical heterogeneity remain unclear but might stem from differences in neurological reserve or thresholds. Some people primarily experience early clinical relapses, others develop asymptomatic, localized inflammatory MRI lesions that come and go [2], while others gradually accrue disability from the outset without remission [3]. Ongoing research is uncovering how one's biological profile influences their clinical presentation.

The biological basis of a clinical relapse involves improper immune cell activation and interaction in the periphery, followed by trafficking of B and T cells across the blood-brain-barrier. This leads to the formation of focal perivenular lesions composed of inflammatory B and T cells, plasma cells, and macrophages in the white matter [4]. These active lesions are visible on gadolinium-enhanced MRI during acute relapses. The acute inflammatory process lasts weeks to months, but some lesions may evolve into chronic active lesions. One form of chronic active lesion is the slowly expanding lesion (SEL), visible on T1 and T2-weighted MRI. Certain SELs progress to paramagnetic rim lesions (PRLs), which is visible on susceptibility-weighted MRI, and pathologically characterized by a demyelinated core surrounded by a rim of activated microglia and macrophages containing myelin debris. The presence of SELs and PRLs is associated with increased clinical disability and is more frequently observed in progressive MS compared to relapsing MS [5].

The biological basis of progressive MS includes at least two major mechanisms– compartmentalized inflammation and neurodegeneration. Compartmentalized inflammation is defined by the establishment of an inflammatory microenvironment in various CNS compartments, which can include the meninges, ependyma, subpial cortex, and deep grey matter, often undetectable by current MRI techniques and primarily observed post-mortem [6]. In both white and grey matter regions, ongoing compartmentalized inflammation is largely driven by innate immune signaling by CNS resident cells like microglia and astrocytes, among others [[7], [8], [9], [10]]. The innate arm of the immune system consists of physical barriers, pattern recognition receptor proteins, and cellular components that discriminate foreign from familiar substances and instruct the adaptive immune system to respond [11]. There is likely interaction and interplay between elements of the innate and adaptive immune systems in neuroinflammation and neurodegeneration. In secondary progressive MS (SPMS) and primary progressive MS (PPMS), B cell aggregates, organized into lymphoid follicle-like structures in the meninges, are associated with activated microglia, neuronal damage, and cortical demyelination. These features are not typically observed in relapsing MS (RMS) [12,13]. The other hallmark of progressive disease pathology is neurodegeneration, which is likely initially due to demyelination of axons, leading to axonal injury [14]. There is likely also an ongoing neuroaxonal loss exacerbated by compartmentalized inflammation mediated by reactive astrocytes and activated microglia [8], mitochondrial dysfunction leading to energy failure and inadequate compensatory mechanisms [15,16].

Current highly effective disease modifying therapies (DMTs), such as monoclonal antibodies targeting CD20, α4-integrin, and CD52, reduce relapse rates by suppressing peripheral immune activity. These therapies are effective in preventing acute, focal white matter inflammation, as measured by clinical relapse or new, expanding, or contrast-enhancing MRI lesions. They are ineffective at reducing clinical markers of progressive disease, which include EDSS worsening and brain volume changes [[17], [18], [19]]. Because the current repertoire of DMTs target peripheral immune mechanisms, they most likely do not also target the critical underlying mechanisms of compartmentalized inflammation and neurodegeneration. Many of these also do not cross the blood-brain barrier. Consequently, many pwMS continue to experience worsening symptoms and clinical deterioration despite the presence of relapse freedom and MRI stability.

There are several explanations for subjective or objective worsening. First, disability can accumulate stepwise due to incomplete recovery after a relapse, known as relapse-associated worsening (RAW). Second, deconditioning can lead to regression to a previous level of disability, often due to inactivity or lack of physical or occupational therapy. Third, there may be gradual accrual of disability without relapse, termed progression independent of relapse (PIRA). Some refer to PIRA as silent progression or smoldering MS, although definitions vary. Many clinical trials and cohort studies define PIRA by EDSS point accumulation in the absence of clinical relapse, though this often overlooks radiological activity, non-motor symptoms, and patient-reported outcomes [20]. Maintaining clarity and consistency in identifying disease progression is critical for developing therapies that effectively target non-relapsing progressive biology – a major unmet need in the field of MS treatment.

## Methods

A comprehensive search of databases, including PubMed and Google Scholar, was performed from 2004 to 2024. The search strategy combined terms related to “multiple sclerosis,” “relapsing MS,” “progressive MS,” “neurodegeneration,” “B and T cells,” “microglia,” “BTK inhibitor,” and “remyelination.” Reference lists of relevant articles were reviewed to identify additional studies. Evidence on novel BTK inhibitors presented at the 2024 European Committee for Treatment and Research in Multiple Sclerosis (ECTRIMS) Conference was also reviewed. Articles were included if they provided insights into understanding the biological and clinical heterogeneity in MS, clarifying the biological mechanisms that drive relapse and progression, highlighting the limitations of current therapies, and investigating the role of BTK inhibition in neuroinflammation and neurodegeneration. As a narrative review, this review is subject to selection bias due to the authors’ judgment of relevance. This review does not intend to systemically evaluate the quality of included studies, but rather to provide a broad overview of the available literature on the topic of BTK inhibitors.

## B cells and BTK

B cells play dual roles in MS, contributing through both pro- and anti-inflammatory mechanisms. Originating from hematopoietic stem cells in the bone marrow, B cells progress through developmental stages via internal mechanisms such as immunoglobulin gene rearrangement, as well as interactions with other cells and antigens. Their development progresses from stem cells to pro-B and pre-B cells in the bone marrow, and then to immature and naïve B cells in the periphery. These lymphocytes mature into memory B cells or, via the plasmablast stage, into plasma cells [21]. These transitions are critical to the immune response and the pathogenesis of MS.

Memory B cells, equipped with antigen-specific receptors, present antigens to T cells. They also amplify the immune response by secreting pro-inflammatory cytokines like TNFα, lymphotoxin-α, and IL-6, which promote T cell proliferation and facilitate the migration of T and B cells across the blood-brain barrier [22,23]. Inside the CNS, B cells interact with T cells and myeloid cells (i.e., monocytes, dendritic cells, macrophages, and microglia). In MS, an imbalance in the pro- and anti-inflammatory properties of myeloid cells drives the ongoing inflammatory response, leading to CNS damage [1].

B cells differentiate into plasma cells, which secrete autoantibodies targeting myelin and other CNS components. Before maturing into plasma cells, B cells become plasmablasts – immature, highly proliferative cells that express CD20, a target for monoclonal antibody therapies in MS. Plasmablasts migrate to inflammatory sites, secrete a modest number of antibodies, and eventually mature into plasma cells, which play a crucial role in adaptive immunity by producing large quantities of antibodies [24]. In MS, plasma cells can generate antibodies both within the CNS and in the peripheral immune system, leading to increased immunoglobulin synthesis. Plasma cells in the CNS secrete antibodies that are detected only in the CSF (and not in peripheral blood) as oligoclonal bands.

On the surface of B cells, two transmembrane protein complexes – Toll-Like Receptor (TLR) and B Cell Receptor (BCR) – function synergistically to mount specific and effective immune responses to pathogens, while maintaining immune tolerance. TLR, part of innate immunity, recognizes broad pathogen patterns and signals dendritic cells, neutrophils, and macrophages to respond. BCR, part of adaptive immunity, detects specific antigens and triggers intracellular signaling to activate B cells, driving their maturation into antibody-secreting plasma cells [25,26]. This intracellular signaling involves several non-receptor tyrosine kinases (NRTKs) which operate within the cytoplasm. Bruton's tyrosine kinase (BTK) is one such critical NRTK expressed in early B lineage cells and myeloid cells [27], but is absent on T cells and plasma cells [28]. Genetic disorders involving the BTK gene, located on the X chromosome, most often dysregulate immune function. One such example is X-linked agammaglobulinemia (XLA), a primary immunodeficiency where pre-B cells in the bone marrow fail to enter circulation, resulting in B cell deficiency and a marked reduction in circulating antibodies [29,30]. In contrast, BTK expression is upregulated in acquired disorders like MS (in chronic active lesions) and in experimental autoimmune encephalomyelitis (EAE), the murine model for MS [31,32].

BTK is a key regulator of early B cell maturation into memory and plasma cells and the B cell proliferative response to antigen. When BCR engages with an antigen, BTK undergoes tyrosine phosphorylation, instigating a cascade of intracellular signaling that influences the activation, homing, proliferation, and survival of B cells [[33], [34], [35]]. BCR-BTK signaling influences integrin activation, and when BTK is inhibited, there is less integrin-mediated immune cell adhesion and migration [35,36]. BTK also mediates calcium signaling and intracellular calcium store depletion in B cells, functions that regulate cell division and growth [37]. While traditionally known for their role in antibody production, B cells also contribute to inflammation through secretion of pro-inflammatory cytokines (IL-6, TNF-α, GM-CSF, and IFN-γ), antigen presentation, and promotion of T cell activation and differentiation. Blocking BTK in the preclinical EAE model decreases expression of molecules (MHCII and CD86) associated with antigen-triggered activation of B cells, cytokine release (IL-6, IFN-γ and IL-10), and reduces T cell proliferation when B cells act as antigen-presenting cells (APCs) and diminishes maturation of naïve T cells into pro-inflammatory Th1 and Th 17 ​cells [38]. By selectively decreasing pro-inflammatory B cell activity, without fully depleting the entire B cell population, BTK inhibitors may spare immunoglobulin-producing plasma cells [[39], [40], [41]].

These findings highlight BTK as an important mediator of systemic inflammation. In MS, autoreactive immune cells in the periphery initiate CNS inflammation. Therefore, BTK is an attractive target for therapeutic intervention.

## BTK in Hematopoietic Myeloid Cells

Myeloid cells can be broadly classified as hematopoietic, originating from and replenished throughout life by the bone marrow, or non-hematopoietic, arising from alternative progenitor sources. Myeloid cells of hematopoietic origin include granulocytes, circulating monocytes, tissue-resident macrophages, mast cells, megakaryocytes, and erythrocytes [42].

In myeloid cell populations, BTK influences normal TLR signaling pathways and activity of Fc receptors (FcR), proteins expressed on the surface of immune cells that allow recognition of antibody-coated targets [36,43,44]. BTK plays a pivotal role in Fc receptor (FcR)-mediated phagocytosis in macrophages [45]. An *ex vivo* study with BTK knock-out mice found BTK to be crucial for recruitment of pro-inflammatory macrophages (defined by intermediate F4/80, high CD11b, and high Ly6C expression) to the peritoneum when treated with bacterial lipopolysaccharide. They also showed that, in BTK-deficient mice, macrophages tended to polarize into an immunosuppressive phenotype (defined by high F4/80, high CD11b, CCL2, CCL17 and CCL22 expression), suggesting that BTK modulates macrophage functional state during inflammation [46]. BTK also regulates mast cell functions, including degranulation and cytokine secretion [47]*.* Additionally, BTK plays a regulatory role in the NLRP3 inflammasome, a protein complex that functions as part of the innate immune systems, in neutrophils [48].

The role of BTK in other myeloid cell types is not well understood. Some evidence suggests that BTK may influence phagocytosis [49] and TNFα production triggered by Fc receptor activation in peripheral monocytes [50]. However, monocytes and polymorphonuclear cells in patients with XLA retain normal receptor expression and exhibit functional phagocytosis, despite lacking BTK [51]. Another study demonstrated that BTK inhibition reduced CD80 and CD86 expression in monocytes; however, monocytes were identified by CD11b, a cell surface marker not restricted to monocytes [38]. Monocytes are typically identified by combinations of CD14 and CD16 surface markers and may be further characterized by others such as HLA-DR, CD64, or CD11b, but these markers are expressed by many myeloid cells and not exclusive to monocytes [52].

## BTK in Non-Hematopoietic Myeloid Cells (Microglia)

BTK is also expressed by tissue-resident macrophages in the CNS, including microglia [53]. Ontogenically distinct from myeloid cells of hematopoietic origin, microglia originate from the extra-embryonic yolk sac [54] and migrate to the CNS, where they self-replenish [55,56]. Microglia express unique markers, including the canonical TMEM119 [32,57], as well as P2RY12 [58]. These markers distinguish microglia from other CNS-resident macrophages.

BTK signaling is involved in regulating microglial proliferation and functional activity, particularly in response to injury. One study reported increased *in vivo* microglial proliferation in knock-in mice expressing a constitutively active form of BTK. Treatment with a BTK inhibitor effectively reduced this proliferative response [59]. Another study showed that blocking BTK signaling in an *ex vivo* rodent model of Alzheimer Disease led to reduced microglial phagocytic activity without altering microglia density [53].

In a different type of injury model, cerebral hypoperfusion led to increased number and expression of BTK in white matter Iba1+ cells, and treatment with tolebrutinib reduced BTK expression and the number of activated Iba1+ cells [60]. This could represent both microglia and other CNS-resident macrophages [59]. In a cuprizone induced mouse model of demyelination, increased BTK expression was found in Iba1+ cells in the corpus callosum [61].

BTK may play a role in the development of demyelinating MS lesions. BTK gene expression is elevated in active and chronic active white matter lesions containing CNS macrophages (defined by MHCII) in post-mortem brain samples from individuals with progressive MS compared to healthy controls [62]. Another study using postmortem brain samples from individuals with MS found increased BTK expression in CD68^+^ myeloid cells that correlated with iron accumulation within active lesions and the rims of chronic active lesions. In the same study, BTK inhibition with evobrutinib downregulated receptors LRP-1 and TIM-1 in CD68^+^ myeloid cells *in vitro* [63]. Therefore, BTK may contribute to MS lesions, potentially through its involvement with CNS macrophages.

Additionally, reduced BTK signaling in CNS-resident macrophages could potentially influence oligodendrocyte and myelin regeneration. One recent study tested the effect of BTK inhibitor treatment following toxin-induced demyelination in two models - *in vivo* transgenic *Xenopus* tadpoles and *ex vivo* murine cerebellar slices. They found that treatment with a BTK inhibitor post-demyelination led to higher numbers of oligodendrocytes or myelin sheaths [64]. This suggests that targeting BTK signaling in the CNS could affect compartmentalized inflammation and possibly remyelination or repair mechanisms in MS.

## BTK Inhibitors

BTK inhibitors are small molecules categorized by their binding mode to BTK as either noncovalent reversible or covalent irreversible. Irreversible inhibitors generally exhibit stronger binding affinity and prolonged activity, resulting in enhancing drug potency. Reversible inhibitors, by recognizing and interacting with specific features of BTK, offer enhanced selectivity and may reduce off-target effects. Drug selectivity is further influenced by the half-maximal inhibitory concentration (IC_50_), which measures the concentration needed to inhibit 50 ​% of BTK activity, guiding therapeutic efficacy [65].

Ibrutinib, the first-generation BTK inhibitor, was approved for treating B cell malignancies including chronic lymphocytic leukemia, small lymphocytic lymphoma, mantle cell lymphoma, marginal zone lymphoma, and Waldenstrom macroglobulinema [66]. Second-generation BTK inhibitors – acalabrutinib, zanubrutinib, and pirtobrutinib – offer more targeted action with a better safety profile. Their success in targeting BCR-BTK signaling pathways in these conditions has spurred the development of BTK inhibitors for other diseases where BTK plays a pivotal role. Currently, more than thirty BTK inhibitors are in various stages of development, many focusing on autoimmune and inflammatory diseases. Six BTK inhibitors under development for MS include evobrutinib, tolebrutinib, fenebrutinib, remibrutinib, orlabrutinib, and BIIB091 (see Table 1).

**Table 1:** Comparison of BTK inhibitors in various phases of clinical development. Each row represents a drug, separated by trial. Each column outlines key attributes and trial data. Abbreviations: IC_50_ (half-maximal inhibitory concentration); ARR (Annualized Relapse Rate); T1 Gd+ (T1 gadolinium-enhancing); EDSS (Expanded Disability Status Scale); OLE (Open Label Extension); AE (Adverse Events); CDP (Confirmed Disability Progression); CDI (Confirmed Disability Improvement); PIRA (Progression Independent of Relapse Activity); NfL (Neurofilament Light Chain); PRLs (Paramagnetic Rim Lesions); SELs (Slowly Expanding Lesions); UTI (Urinary Tract Infection); NEDA-3 (No Evidence of Disease Activity-3); SDMT (Symbol Digits Modalities Test); T25FW (Timed 25-Foot Walk); 9HPT (9-Hole Peg Test); MSIS-29 (Multiple Sclerosis Impact Score); CVLT-II (California Verbal Learning Test II); MSQoL-54 (Multiple Sclerosis Quality of Life-54).

**Drug**	**Mode of Binding**	**Half-Life, IC_50_ Activity**	**Comparator**	**Trial Name, Phase,**ClinicalTrials.gov**Identifier**	**Relapsing Endpoints**	**Progressive Endpoints**	**Other Outcomes**

	Irreversible, covalent	2 ​h, 33.5 ​nM	Placebo	Phase II, NCT02975349	ARR: No difference T1 Gd ​+ ​lesions: Reduced in 75 ​mg QD evobrutinib	EDSS: No difference	OLE: ARR 0.11; EDSS stable AE: Nasopharyngitis, liver enzyme elevations
**Evobrutinib**			Teriflunomide	evolutionRMS1, Phase III, NCT04586010, evolutionRMS2, Phase III, NCT04586023	ARR: No difference New/enlarging T2 lesions or Gd ​+ ​T1 lesions: No difference NfL: No difference in evolutionRMS1, slighter reduced in evolutionRMS2	CDP, CDI, PIRA: No difference Brain, cortical grey, thalamic volume; PRLs: No difference Small differences in SEL volume and black hole rate favoring evobrutinib.	SDMT, PROMIS Fatigue score: No difference AE: Elevated liver enzymes, COVID-19, headache, nasopharyngitis
	Irreversible, covalent	2 ​h, 0.7 ​nM	Placebo	Phase IIb, NCT03889639	T1 Gd ​+ ​lesions: Reduced in tolebrutinib (dose-dependent)		OLE: ARR 0.23, EDSS stable
			Placebo	Phase II, NCT04742400		PRLs: No difference	
**Tolebrutinib**			Teriflunomide	GEMINI 1, Phase III, NCT04410978 GEMINI 2, Phase III, NCT04410991	ARR: No difference Higher T1 Gd ​+ ​lesions in tolebrutinib. New/enlarging T2 lesions: No difference	CDP: Reduced by 29 ​% in pooled 6-month analysis. CDI: No difference Brain volume: No difference	AE: Liver enzyme elevations, COVID-19, nasopharyngitis, headache
			Placebo	HERCULEUS, (SPMS) Phase III, NCT04411641	New/enlarging T2 lesions: Reduced by 38 ​% in tolebrutinib	CDP: Reduced by 31 ​% in tolebrutinib CDI: 88 ​% improvement (10 ​% in tolebrutinib compared to 5 ​% in placebo) Brain volume: No difference	AE: Liver enzyme elevations (one liver transplant case), COVID-19, UTI
			Placebo	PERSEUS, (PPMS) Phase III, NCT04458051	New/enlarging T2 lesions, NfL level: Results pending	CDP, CDI, brain volume: Results pending	SDMT, CVLT-II, MSQoL-54, immunoglobulin level, and chitinase-3 like protein 1 to be assessed.
**Fenebrutinib**	Reversible, noncovalent	4.2–9.9 ​h, 2.9 ​nM	Placebo	FENopta, Phase II, NCT05119569	T1 Gd ​+ ​lesions and new/enlarging T2 lesions: ≥90 ​% reduction in fenebrutinib		OLE: ARR 0.04, EDSS stable AE: Liver enzyme elevations, headache, nasopharyngitis, abdominal pain, COVID-19, UTI
			Teriflunomide	FENhance 1, Phase III, NCT04586010 FENhance 2, Phase III NCT04586023	ARR, T1 Gd ​+ ​lesions, new/enlarging T2 lesions, and NfL level: Results pending	CDP, brain volume: Results pending	MSIS-29 and SDMT to be assessed.
			Ocrelizumab	FENtrepid, Phase III, NCT04544449	NfL level: Results pending	CDP, brain volume change: Results pending	MSIS-29 and SDMT to be assessed.
**Remibrutinib**	Irreversible, covalent	1–2 ​h, 1.3 ​nM	Teriflunomide	REMODEL-1, Phase III, NCT05147220 REMODEL-2, Phase III, NCT05156281	ARR, time to 1st confirmed relapse, T1 Gd ​+ ​lesions, and new/enlarging T2 lesions, NEDA-3, NfL level: Results pending	CDP, CDI: Results pending	SDMT, T25FW, 9HPT, and MSIS-29 to be assessed.
**Orelabrutinib**	Irreversible, covalent	4 ​h, 1.6 ​nM	Placebo	Phase II, NCT04711148	90.4 ​% reduction in new T1 Gd ​+ ​lesions (12-week interim analysis). Full results pending. ARR: Results pending		Safety and tolerability also to be assessed.
**BIIB091**	Reversible, noncovalent	0.8 ​h, 87 ​nM	Diroximel fumarate	FUSION, Phase II, NCT05798520	T1 Gd ​+ ​lesions, new/enlarging T2 lesions: Results pending		Safety and tolerability also to be assessed.

## Evidence in MS

**Evobrutinib**, an oral BTK inhibitor, found efficacy in reducing disease severity in pre-clinical studies [38]. It forms irreversible covalent bonds, has a 2-h half-life [67], and an IC_50_ potency of 33.5 ​nM [68]. In a double-blind, multi-center, randomized Phase II trial *(NCT02975349),* evobrutinib was tested against placebo in 267 patients with RMS. Participants were assigned to one of five groups: evobrutinib (25 ​mg once daily, 75 ​mg once daily, or 75 ​mg twice daily), open-label dimethyl fumarate, or placebo. Dimethyl fumarate was included as a reference, not a direct comparator. Relapsing clinical endpoints included reduction in gadolinium-enhancing T1 lesions and annualized relapse rate (ARR). At 24 weeks, the 75 ​mg once-daily group showed significantly fewer enhancing lesions compared to placebo, while no significant reduction was observed in the 25 ​mg or 75 ​mg twice-daily groups. There was no significant difference in ARR. Additionally, the progressive clinical endpoint of change in Expanded Disability Status Scale (EDSS) was examined; there were no significant differences in EDSS change [69]. After week 24, in an open-label extension (OLE), the placebo group switched to evobrutinib 25 ​mg, with follow-up continuing for 48 weeks. ARR was 0.11 and EDSS scores remained stable in the 128 individuals who completed OLE treatment. The most common adverse events (AEs) in the evobrutinib group were nasopharyngitis and asymptomatic, reversible liver enzyme elevations [70].

Two double-blind, randomized Phase III trials, *evolutionRMS 1* and *2* compared evobrutinib to teriflunomide in 1124 participants with RMS were treated for up to 156 weeks. Relapsing clinical endpoints included ARR, number of gadolinium-enhancing T1 lesions, number of new or enlarging T2 lesions, and serum neurofilament light chain (NfL) concentration at week 12. Results demonstrated evobrutinib and teriflunomide achieving respective ARRs of 0.15 versus 0.14 in *evolutionRMS 1* and 0.11 versus 0.11 in *evolutionRMS 2*, indicating no added benefit to evobrutinib in reducing clinical relapse. Interestingly, the total number of gadolinium-enhancing T1 lesions was numerically higher for evobrutinib than for teriflunomide in both studies. There was no significant difference in the number of new or enlarging T2 lesions between groups. Mean serum NfL concentrations were similar between evobrutinib and teriflunomide in evolutionRMS1 and slightly reduced in the evobrutinib group in evolutionRMS2 [71]. Several factors could explain the lack of efficacy differences. Teriflunomide performed unusually well as a comparator^1^ with ARR results lower than typically observed in previous clinical trials (0.32–0.37) [72,73]. Real-world studies report ARR as low as 0.11 to 0.22, although real-world populations and settings, baseline disease activity, and duration of observation vary [[74], [75], [76]]. The ARR in RMS patients on platform injectables or low-moderate efficacy oral therapies ranges from 0.16 to 0.39 [[77], [78], [79], [80]]. A post-hoc analysis suggests the increase in enhancing lesions and similarity of ARRs may be driven by a subset of younger patients with higher baseline inflammatory disease; these data are unpublished [71]. Additionally, evobrutinib's Phase II trial did not show a significant reduction in ARR compared to placebo, suggesting either a mechanistic limitation or that ARR may not fully capture the drug's therapeutic benefit, despite promising MRI outcomes.

Progressive clinical endpoints included 12- and 24-week confirmed disability progression (CDP), 24-week confirmed disability improvement (CDI), as well as exploratory endpoints of PIRA (defined as 12-week CDP and 12-week confirmed worsening (EDSS, 9-Hole Peg Test and Timed 25-foot Walk)). Other progressive outcomes included slowly expanding lesions (SELs); change in brain, cortical grey matter, and thalamic volume; rate of new hypointense T1 gadolinium-enhancing lesions (“black holes”), and paramagnetic rim lesions (PRLs). Additional outcomes included Symbol Digit Modalities Test (SDMT) and patient-reported PROMIS Fatigue score. Results showed no significant difference in CDP or CDI between groups in both studies. PIRA, SDMT, and PROMIS Fatigue score were similar between groups. Overall, the MRI outcomes were unimpressive with small differences in SEL volume and rate of new black holes favoring evobrutinib in both studies. No significant differences were found in brain, cortical grey matter, and thalamic volume loss, and PRLs between groups [72, appendix].

Safety data were similar between the two groups, though evobrutinib had a slightly higher incidence of elevated liver enzymes, which normalized after discontinuation of evobrutinib. Common AEs included COVID-19, headache, asymptomatic elevated liver enzymes, and nasopharyngitis.

**Tolebrutinib** is an oral BTK inhibitor with irreversible, covalent binding, a half-life of 2 ​h [81] and an IC_50_ potency of 0.7 ​nM [68]. There is limited data on the relative CNS penetrance of BTK inhibitors in development for MS [68].Tolebrutinib has been assessed in three Phase II trials and is currently under evaluation in four Phase III trials. In a Phase IIb trial (*NCT03889639*), 129 individuals with RMS were treated over 12 weeks and evaluated on clinical relapsing measures of total number of gadolinium-enhancing T1 lesions and new/enlarging T2 lesions. Results demonstrated a dose-dependent reduction in gadolinium-enhancing T1 and new/enlarging T2 lesions compared to placebo, with 60 ​mg being the most effective dose [82]. The OLE *(NCT03996291)* included 82 ​% of participants and explored clinical measures of ARR and EDSS change. Overall, the ARR was 0.23, below baseline, and EDSS remained stable after 2.5 years of treatment [83].

A small Phase II study *(NCT04742400)* evaluated whether tolebrutinib could affect PRLs in MS patients. Seven patients switched from baseline anti-CD20 therapies to tolebrutinib, while six continued on anti-CD20 therapies for 96 weeks. The study found no significant changes in the size or number of PRLs [84].

Two double-blinded, randomized Phase III trials (*GEMINI 1 and 2)* evaluated tolebrutinib in RMS with clinical relapsing endpoints of ARR, new/enlarging T2 lesions, and gadolinium-enhancing T1 lesions. Over 96 weeks, 1873 subjects (mean age 37 and 67 ​% female) received either tolebrutinib or teriflunomide. Mean baseline EDSS was 2.4 and mean time since MS onset was 4.3 years, with 63 ​% having no prior DMT exposure. Tolebrutinib did not significantly reduce ARR compared to teriflunomide. In *GEMINI 1 (NCT04410978)*, ARRs were 0.13 for tolebrutinib and 0.12 for teriflunomide. In *GEMINI 2 (NCT04410991),* both groups had an ARR of 0.11. A pooled six-month analysis showed identical ARRs (0.12) for both groups. While tolebrutinib did not demonstrate superiority over teriflunomide in suppressing relapses, it may offer comparable efficacy. The tolebrutinib group had significantly more gadolinium-enhancing T1 lesions and no difference in T2 lesions. These MRI findings are particularly intriguing, as they may indicate that tolebrutinib changes something about the natural evolution of focal, inflammatory lesions. Clinical progressive endpoints included CDP, CDI, and brain volume changes. A pooled six-month analysis reported tolebrutinib showing a 29 ​% reduction in the risk of time to 6-month CDP and no difference in CDI. Brain volume changes were similar between the two groups. AEs included elevated liver enzymes, COVID-19, nasopharyngitis, and headache in both groups. Elevated liver enzymes (≥3x upper limit of normal) were noted in 5.6 ​% of tolebrutinib users and 6.3 ​% of teriflunomide users, with all cases resolving without complications [85].

The Phase III *HERCULEUS* trial *(NCT04411641)* tested tolebrutinib against placebo in individuals with non-relapsing SPMS and analyzed progressive endpoints of 6-month CDP and CDI, as well as brain volume changes. The trial enrolled 1131 participants ages 18–60 (mean age 49) with SPMS diagnosis, baseline EDSS 2.0–6.5, evidence of disability progression in the past year, and no clinical relapses for the preceding two years. At baseline approximately 40 ​% had an EDSS ≤5.5. Mean time since the onset of RMS was about 17 years, with a mean of 7 years since the most recent relapse. The clinical relapsing outcome was number of new/enlarging T2 lesions. Tolebrutinib reduced new/enlarging T2 lesions by 38 ​%, a statistically significant result. In addition, after 48 months, tolebrutinib achieved its primary endpoint (a progressive clinical outcome) in delaying time to 6-month CDP by 31 ​% compared to placebo, a promising result for progressive disease. Additionally, 10 ​% of participants on tolebrutinib experienced a 6-month CDI, compared to 5 ​% on placebo, reflecting an 88 ​% benefit. Tolebrutinib reduced new/enlarging T2 lesions by 38 ​%, a statistically significant result. There was no difference in brain volume loss between the groups. AEs included elevated liver enzymes (≥3x upper limit of normal) in 4.1 ​% of the tolebrutinib group versus 1.6 ​% in placebo during the first three months. In the tolebrutinib group, 0.5 ​% had peak elevations ≥20x upper limit of normal, with most resolving without sequalae. One participant, however, required a liver transplant and later died from post-operative complications, prompting study protocol revisions for more frequent monitoring in the first 90 days. Other common AEs included similar rates of COVID-19 and urinary tract infections in both [86,87].

The fourth Phase III trial, *PERSEUS (NCT04458051)*, is evaluating tolebrutinib versus placebo in 990 adults aged 18–55 with PPMS. This trial focuses on the progressive endpoint of delay in disability progression over up to five years, with results expected in 2025. Due to reports of elevated liver enzymes in some participants, the FDA placed a partial hold the U.S. trials, although recruitment and treatments continue in other countries with enhanced safety monitoring [88].

**Fenebrutinib** is an oral BTK inhibitor with reversible, noncovalent binding, a half-life of 4.2–9.9 ​h [89], and an IC_50_ potency of 2.9 ​nM [68]. It was evaluated in the double-blind, multi-center, randomized Phase II trial *FENopta (NCT05119569)* on the clinical endpoint of relapsing disease, a reduction in gadolinium-enhancing T1 lesions. There were 109 adults aged 18–55 with RMS randomized to fenebrutinib or placebo for 12 weeks, followed by an OLE for up to 48 weeks. In the fenebrutinib and placebo arms, 25 ​% and 47 ​% had prior DMT use, respectively. A secondary end point was the reduction in new or enlarging T2 lesions. After 12 weeks, fenebrutinib showed a ≥90 ​% reduction in gadolinium-enhancing and new or enlarging T2 lesions relative to placebo. AEs occurred in 38 ​% of the fenebrutinib group and 33 ​% of the placebo group, with the most common AEs in fenebrutinib being elevated liver enzymes, headache, nasopharyngitis and upper abdominal pain [90]. In the OLE, results of relapsing endpoint showed that 99 ​% of participants were free from new gadolinium-enhancing lesions and had an ARR of 0.04. The progressive endpoint, EDSS, remained stable. The safety profile remained consistent, with the most common AEs being urinary tract infection, COVID-19, and pharyngitis [91].

Fenebrutinib is currently being evaluated in three ongoing Phase III trials. *FENhance 1 (NCT04586010)* and *2* (*NCT04586023)* are enrolling approximately 1500 participants with RMS, comparing fenebrutinib or teriflunomide. Relapsing outcomes include ARR, gadolinium-enhancing T1 lesions, and new/enlarging T2 lesions. Progressive outcomes include time to CDP and brain volume changes [92,93]. *FENtrepid (NCT04544449)* is assessing fenebrutinib in 985 adults with PPMS, compared to ocrelizumab. This trial focuses on progressive outcomes of time to CDP and brain volume changes, as well exploratory patient-reported outcomes [94,95]. This is particularly noteworthy in the field of MS for its comparison with a highly effective therapy and the only FDA-approved treatment for PPMS. Results from these trials are anticipated between 2025 and 2026.

**Remibrutinib** is an irreversible covalent oral BTK inhibitor, with a half-life of 1–2 ​h [96], and an IC_50_ potency of 1.3 ​nM [97]. It has been studied in other autoimmune conditions and is now being evaluated in RMS. Two Phase III double-blind, randomized trials *REMODEL-1 (NCT05147220) and 2* (*NCT05156281)* are actively recruiting 1600 adults to compare remibrutinib with teriflunomide [98,99]. Relapsing outcomes include ARR, gadolinium-enhancing lesions, new or enlarging T2 lesions, and NfL level. Progressive outcomes include time to CDP and CDI. The trial is set to last up to 30 months, with an optional OLE of up to 5 years, and completion expected in 2030.

**Orelabrutinib** is an irreversible covalent oral BTK inhibitor with a half-life of 4 ​h [100] and an IC_50_ potency of 1.6 ​nM [101] being evaluated in a double-blind, randomized Phase II trial (*NCT04711148*) involving 160 adults with RMS. Participants were randomized to receive 50 ​mg daily, 80 ​mg daily, or 50 ​mg twice daily orelabrutinib, or placebo for 24 weeks with an optional OLE. The trial evaluated relapsing endpoints of cumulative number of gadolinium-enhancing lesions and ARR, as well as assess safety and tolerability. A 24-weeks interim analysis showed that all treatment groups had statistically significant control of new gadolinium enhancing lesions, with the 80 ​mg dose achieving the highest efficacy with a 90.4 ​% relative reduction in cumulative number of new lesions. Full results are expected in 2026 [102,107].

Lastly, **BIIB091** is a reversible, noncovalent oral BTK inhibitor with a half-life of 0.8 ​h and an IC_50_ potency of 87 ​nM [103]. BIIB091 is being tested in the *FUSION* trial *(NCT05798520),* a double-blind randomized Phase II trial comparing BIIB091 with diroximel fumarate in 275 adults with RMS over 48 weeks. Following evaluations for safety and tolerability, the study will assess the relapsing outcome of cumulative number of gadolinium-enhancing lesions. Recruitment is ongoing, with results anticipated in 2026 [104].

Additional details about the studies are available at clinicaltrials.gov.

## BTK Degraders

BTK inhibitors have significantly advanced the treatment of B cell malignancies and show potential in immune-mediated disorders. However, some patients with B cell malignancies develop resistance to BTK inhibitors due to mutations that allow BCR signaling to persist. To address this, novel molecules known as BTK degraders are being developed to target both wild-type and mutant forms of BTK, marking it for degradation and destruction [105,106]. These degraders may offer a valuable therapeutic option alternative for patients who are unresponsive to BTK inhibitors.

## Discussion

BTK inhibitors represent a promising emerging class of therapeutics with the potential to address several unmet needs in MS treatment. While current anti-CD20 therapies deplete circulating B cells and effectively suppress clinical relapses and new focal MRI lesions, they are not designed to target chronic, compartmentalized inflammation that underlies progressive MS. Additionally, long-term peripheral B cell depletion, including plasma cells responsible for immunoglobin production, may increase the risk for serious infections, malignancy and reduce vaccine effectiveness.

In contrast, BTK inhibitors offer a unique dual mechanism of action, potentially targeting both B cells in the periphery and microglia in the CNS. Both are key players in MS pathology, though further research on the role of BTK inhibition in microglia and reliable methods for studying microglia is critical. Selective reduction of pathogenic B cell activity, rather than total B cell depletion, may allow for continued immunoglobulin production and increased preservation of adaptive immune function. Moreover, the small molecular size is better for penetration of the blood-brain-barrier, potentially targeting B cells within the CNS, as well as targeting myeloid cells contributing to compartmentalized inflammation.

Thus far, the clinical trial results for BTK inhibitors are mixed. While it may be tempting to directly compare outcomes across studies, this should be avoided due to variations in study design, patient populations, and endpoints, all of which can significantly influence results. It is important to recognize the distinct objectives of Phase II and Phase III trials. Phase II trials primarily aim to evaluate the safety and preliminary efficacy of a therapy to determine its suitability for larger-scale testing. In contrast, Phase III trials are comprehensive, large-scale studies designed to confirm therapeutic efficacy, establish long-term safety, and provide critical data for regulatory bodies to assess the overall risk-benefit profile of a drug.

Phase II studies have generally shown that BTK inhibitors have an acceptable safety profile, although concerns regarding liver toxicity have emerged. Trials for fenebrutinib and tolebrutinib showed promising reductions in MRI markers of acute inflammatory activity, suggesting that BTK inhibitors may influence relapse biology. The selection of teriflunomide as a comparator is strategic, offering a reasonable benchmark for evaluating the efficacy, safety, and convenience of these novel therapies.

Phase III studies have provided more comprehensive data on the safety, tolerability, and efficacy of BTK inhibitors. Although elevated liver enzymes have led to enrollment pauses and increased monitoring, these elevations have generally been asymptomatic and reversible upon treatment discontinuation. If approved for MS, BTK inhibitors will likely necessitate regular lab monitoring to detect potential drug-induced liver toxicity. Infections reported have typically been mild to moderate, predominantly upper respiratory in nature. Importantly, BTK inhibitors may pose a lower risk of severe infections compared to current highly effective therapies, as they do not cause full B cell depletion, preserving some immune function. The *FENtrepid* trial is expected to offer valuable safety data by comparing this new class of drugs to the widely used anti-CD20 therapy, ocrelizumab.

Phase III data show that evobrutinib and tolebrutinib do not surpass teriflunomide in terms of suppressing clinical relapses. They appear to demonstrate similar efficacy. It is worth noting, however, that the duration of these Phase III trials may have been too short to reveal a more substantial effect of BTK inhibition on relapse rates. Interpreting the effects on focal MRI lesions is more complex due to variability in lesion evolution. MRI does not capture every stage of lesion evolution, and further data are needed to understand how BTK inhibition influences lesion development. In contrast to the clinical and imaging endpoints for relapse biology, the outcomes that are related to progressive disease are compelling. For example, tolebrutinib outperforms teriflunomide in both relapsing MS and non-relapsing SPMS, suggesting that it also may target mechanisms of progressive biology. In fact, individuals with non-relapsing MS experienced improvements in disability. If BTK inhibitors exert a pronounced influence on progressive disease-mediating microglia, then their use may lead to a greater impact on progressive disease outcomes.

The MS community eagerly anticipates the results from Phase III trials on fenebrutinib, with hopes that BTK inhibition modifies the course of progressive disease, and possibly relapsing forms as well. In the coming years, we will learn of the trial results for Remibrutinib, Orelabrutinib, BIIB091 and potentially others. A critical question for these Phase III trials is whether BTK inhibitors are efficacious across diverse patient populations, particularly in those with progressive disease, where therapeutic options remain scarce. BTK inhibition may target key aspects of compartmentalized inflammation via microglia. Each person with MS possesses a unique biological and clinical profile shaped by factors such as genetic susceptibility, environmental risks, immune signatures, neuroimaging and serum biomarkers, neurological reserve, disease course and disability trajectories, and treatment responses. Early identification of these characterisitcs will be essential for designing future personalized disease modifying treatment strategies. These strategies could one day involve one or more therapies that each focus on relapse or progressive biology.

## Author contributions

Laura R Naydovich: Conceptualization, Investigation, Roles/Writing - original draft, Writing - review & editing.

Jennifer L Orthmann-Murphy: Investigation, Writing - review & editing.

Clyde E Markowtiz: Conceptualization, Writing - review & editing.

## Declaration of competing interest

The authors declare that they have no known competing financial interests or personal relationships that could have appeared to influence the work reported in this paper.

## Footnotes

1 In an earlier version of the *evolutionRMS 1 and 2* protocols*,* Avonex (interferon beta-1a) was initially chosen as the active comparator. Because of updates in standard of care, patient preference for oral drug over injectable, and current regulatory expectations for rigorous data on experimental drug performance, teriflunomide was deemed a more relevant and competitive comparator for evobrutinib.
